# Seroprevalence of *Toxoplasma gondii* in the Eurasian otter (*Lutra lutra*) in England and Wales

**DOI:** 10.1186/1756-3305-6-75

**Published:** 2013-03-19

**Authors:** Elizabeth A Chadwick, Joanne Cable, Alex Chinchen, Janet Francis, Edward Guy, Eleanor F Kean, Sarah C Paul, Sarah E Perkins, Ellie Sherrard-Smith, Clare Wilkinson, Dan W Forman

**Affiliations:** 1 School of Biosciences, Sir Martin Evans Building, Cardiff University, Cardiff CF10 3AX, UK; 2 Toxoplasma Reference Unit, Public Health Wales Microbiology, Singleton Hospital, Swansea SA2 8QA, UK; 3 School of the Environment and Society, Swansea University, Swansea SA2 8PP, UK

**Keywords:** Wildlife disease, Spatial distribution, Sabin Feldman, Surveillance, Zoonosis

## Abstract

**Background:**

*Toxoplasma gondii* is found on all continents and can infect all endothermic vertebrates. Toxoplasmosis is a globally important zoonosis with potentially devastating health impacts both for humans and a range of domestic and wild species. The World Health Organisation have repeatedly recommended the collection of accurate epidemiological data for *T. gondii*, yet despite recognised links between infection of wildlife, domestic animals and humans, seroprevalence in wild species is rarely monitored. Here, serological investigation using the Gold Standard Sabin-Feldman Dye Test was used to test for *T. gondii* in Eurasian otters (*Lutra lutra*) found dead, mainly as road-kill, in England and Wales. This is the first spatially widespread study of *T. gondii* in UK wildlife, and the first extensive survey of *T. gondii* in Eurasian otters, a sentinel species of fresh waters.

**Findings:**

Infection was both common (39.5% prevalence, n = 271) and widespread, with significantly more infection in the east than the west of the UK. There was an increase in seroprevalence with age, but no sex bias.

**Conclusions:**

The relatively high prevalence of *T. gondii* in a predominantly piscivorous freshwater mammal suggests widespread faecal contamination of freshwater ecosystems with oocysts. Continued surveillance of the Eurasian otter for *T. gondii* is valuable because of conservation concerns due to the otter’s ‘near threatened’ status on the IUCN Red List and because of the host’s role as a sentinel for freshwater health.

## Findings

### Background

*Toxoplasma gondii* is found on all continents, has a wide variety of hosts including humans, and can probably infect all endothermic vertebrates [1,2]. Individuals become infected either by ingestion of oocysts (shed with the faeces of felids, the definitive host) in contaminated water and soil, by ingestion of tissue cysts (in raw or undercooked meat from infected animals), or through congenital transfer [1]. It has also been suggested that human infection can occur after ingestion of tachyzoites in milk [3]. Such reports are rare, however, and the significance of this potential route of infection in other species has not been established. Toxoplasmosis is a globally important zoonosis which can have devastating health effects. A clear understanding of how *Toxoplasma* moves through the environment, between wildlife, domesticated animals and humans, is critical in informing risk assessment and identifying potential interventions to reduce the burden of disease.

Although traditionally considered a parasite of terrestrial habitats, recent reports have identified *T. gondii* in a range of marine (e.g. [4,5]) and freshwater species (e.g. [6,7]). Here we estimate the seroprevalence of *T. gondii* in the Eurasian otter (*Lutra lutra*), a semi-aquatic mustelid. We compare infection status in otters across different regions of the UK, and investigate whether seroprevalence differs with sex or age-class.

### Methods

Otters found dead in England and Wales were collected as part of a national monitoring programme (http://www.otterproject.cf.ac.uk). A detailed post mortem examination was performed on each otter (data not presented) and animals were categorised by sex and age-class (juvenile, sub-adult and adult [8]). Blood samples were collected from the thoracic cavity of 271 otter carcasses between 1995 and 2008 (though only 8 were collected prior to 2004), and were stored in 1.5 ml microfuge tubes (Eppendorf) at −20°C. The predominant cause of death was road traffic accident (90.8%, n = 246). Others died from infection, fighting injuries and/or emaciation (5.5%, n = 15), some were shot or drowned (2.6%, n = 7), and in a few cases cause of death could not be ascertained (1.1%, n = 3).

**Figure 1:**
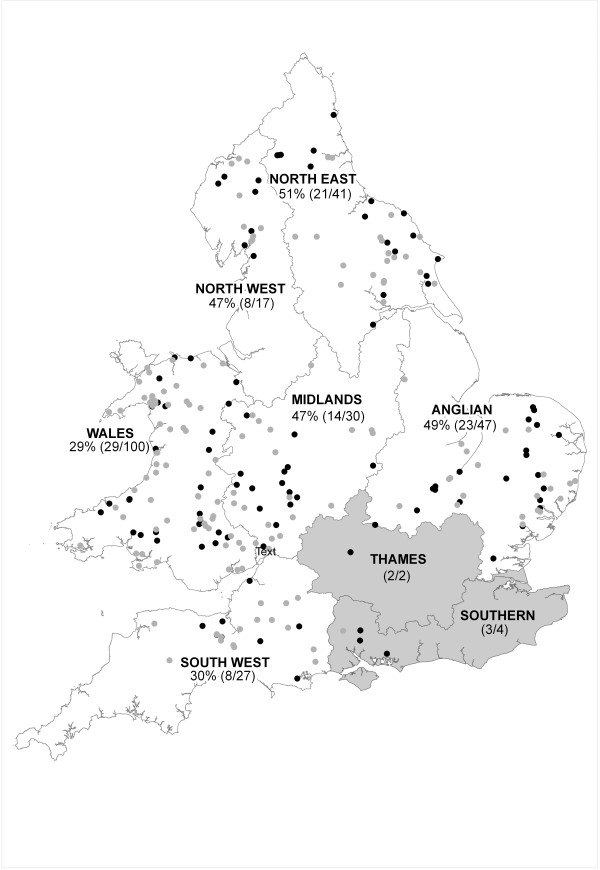
**Spatial variation in *Toxoplasma gondii *seroprevalence in UK otters.** Individual otters tested are shown in black (seropositive) or grey (seronegative). The percentage of Eurasian otters seropositive for *Toxoplasma gondii* is indicated for each of eight Regions (Environment Agency management Regions, based on groups of river catchments), and numbers of seropositive/total number of individuals tested are shown in parentheses. Results for Thames and Southern Regions, shaded grey, were excluded from analyses due to low sample size (n < 5).

Serum samples were analysed for antibodies to *T. gondii* according to the cytoplasm modifying dye test first described by Sabin and Feldman [9], at the National Toxoplasma Reference Unit, Swansea (the established gold standard test for human serodiagnosis, and subject to an accredited national quality assurance scheme [UK National External Quality Assurance Scheme, UKNEQAS]). Blood samples were first diluted 1:4 with saline to ensure readability, due to the degraded nature of many of the samples, and the use of whole blood rather than serum. The cut-off for the assay, of <2 IU/ml, is therefore equivalent to a cut-off titre of 1/8, used here for reporting positive results. The geographic origin of samples across England and Wales was plotted using ArcMap GIS (version 9.2), and categorised by Region (UK Environment Agency management Regions, based on groups of river catchments) (Figure 1). A Generalised Linear Model (GLM), with a binomial error distribution, was fitted to the prevalence data with host sex, age and Region as predictors (n = 262). Year was not included in the model because of unbalanced sampling; between year differences were examined separately for 2004–08. Data were excluded from Thames and Southern Regions due to small sample sizes (n = 2 and 4, respectively) and where location or age data were missing (n = 3). Non-significant terms were removed from the model by ANOVA comparisons and the simplest model chosen using the Akaike Information Criteria (AIC); pairwise comparisons were made for significant factors using parameter contrast within the GLM. All analysis was conducted using R version 2.12.1 [10].

### Results

**Figure 2:**
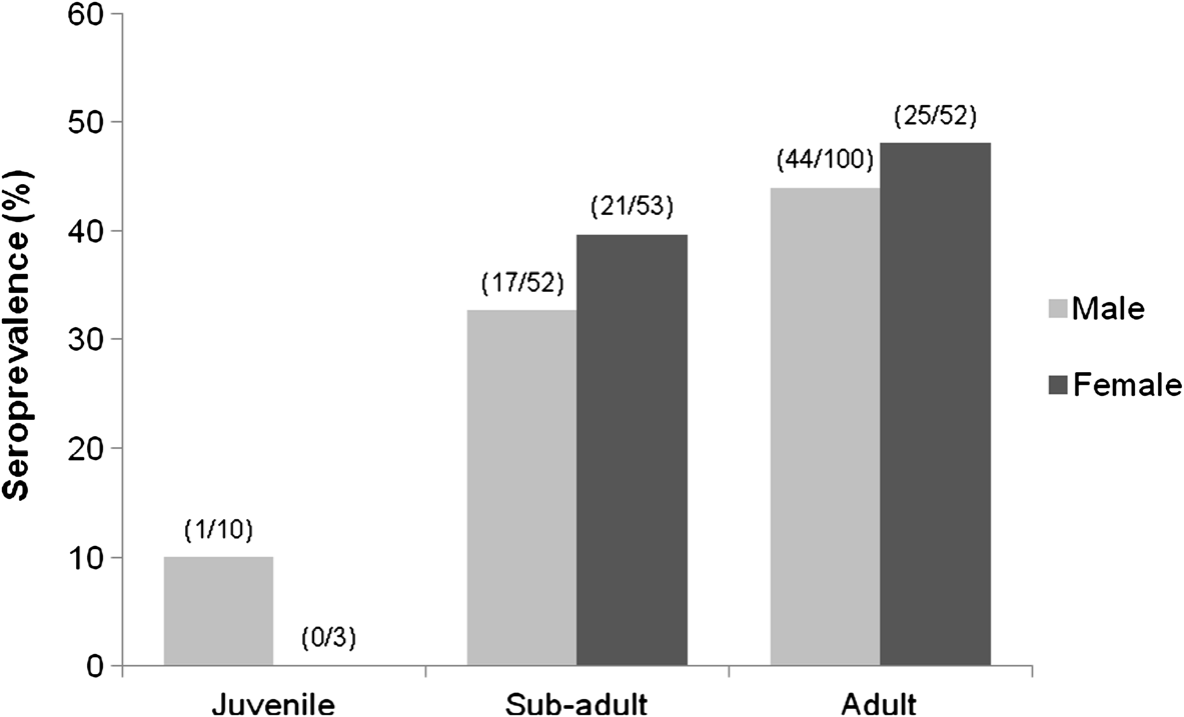
**Individual variation in *Toxoplasma gondii *seroprevalence in UK otters.** The percentage of Eurasian otters seropositive for *Toxoplasma gondii* is indicated for groups of individuals separated by sex and age class. Numbers of seropositive/total number of individuals in each group are shown in parentheses.

Antibodies to *Toxoplasma gondii* were found in 39.9% (108/271) of otters. Positive results were recorded at titres between 1/8 (16%), 1/16 (61%), 1/32 (18%), 1/64 (2%) and 1/125 (3%). Between 2004 and 2008, seroprevalence showed limited variation, between 40 and 44%. Seropositive individuals were widely distributed across England and Wales (Figure 1). Region (GLM: Chi-squared = 10.734, df = 5, n = 262, p = 0.057) and host age class (GLM: Chi-squared = 8.283, df = 2, n = 262, p = 0.016) explained some variation in *T. gondii* prevalence. Contrast analysis showed that prevalence in Wales (29%) was significantly lower than in Anglian (49%) (t_254_ = −2.21, p = 0.028) and North East Regions (51%) (t_254_ = −2.61, p = 0.010), and there was a close to significant difference in prevalence between South West Region (30%) and North East Region (51%) (t_254_ = 1.74, p = 0.084). There were no significant differences in prevalence between other Regions (p > 0.1). Seroprevalence was significantly higher in adults (45%) than juveniles (8%) (t_254_ = 2.13, p = 0.034), with near significant difference between sub-adults (36%) and juveniles (t_254_ = 1.80, p = 0.073). Variation in prevalence was not associated with the sex of otters (Figure 2).

### Discussion

Otters seropositive for *Toxoplasma gondii* were detected across England and Wales, with an overall prevalence nearing 40%. *Toxoplasma* in Eurasian otters has been reported once previously, with positive results in all six Eurasian otters tested from Spain [11]. Although *T. gondii* has been linked to high levels of mortality in sea otters (*Enhydra lutris*) [12], there are no reported cases of *Toxoplasma* related mortality in Eurasian otters. This is difficult to evaluate, however, due to a greater likelihood of finding road killed otters as opposed to those dying of other causes. No signs apparent at post mortem were indicative of *T. gondii* infection, but such signs would be limited to animals with acute, active infection. Our study focuses specifically on prevalence, and so includes individuals with latent infection. The integrity of tissues used in this study (from road kill, frozen prior to examination) precluded the application of alternative diagnostic methods such as immunohistochemistry. In keeping with reports in other mammals (e.g. [7,11]), there was no evidence for a sex bias, but seroprevalence did increase with host age. No change over time was apparent during the period 2004–08, suggesting that infection rates are stable.

Seroprevalence of *T. gondii* was lower in the west of the UK than in the east. To the best of our knowledge there are no other spatially comparable surveys of *Toxoplasma* in wildlife in the UK, although there have been localised studies of badgers in southern England [13], the urban house mouse in Manchester [14], and a widespread but not spatially explicit study of foxes across the UK [15]. It is not clear, therefore, whether the spatial variation reported here is species specific, or whether seroprevalence in otters parallels that in other wild species. Variation in seroprevalence between regions might reflect a variety of factors, including differences in exposure (for example through regional variation in the number of domestic or feral cats, or climatic influence on survival of oocysts in the environment) or differences in host susceptibility or parasite virulence. In contrast to otters, seroprevalence data from human blood donors show higher seroprevalence in the west than the east of the British Isles [16], suggesting species-specific risk factors for infection.

Infection of semi-aquatic species such as the Eurasian otter with *T. gondii* is likely to occur via multiple routes. Eurasian otters in England and Wales feed primarily in freshwater systems, but also prey on marine species in some coastal areas. They travel along water courses, but also across land, often between watersheds, and they use terrestrial holts [17]. Infection may therefore arise through environmental contamination of freshwater, marine or terrestrial systems with oocysts. Although reliant primarily on ectothermic prey, such as fish and amphibians that reportedly do not develop tissue cysts of *T. gondii*, otters are highly opportunistic predators and occasionally take mammals and birds [18]. Endothermic vertebrates were identified in the stomach contents of 10% of 618 UK otters examined post mortem (Cardiff University Otter Project, unpublished data), suggesting exposure via ingested prey items is also likely to occur. However, as is the case in general for *T. gondii* infected species, confirming a specific route of exposure in otters is not possible.

Whether exposure of otters to *T. gondii* is predominantly via oocysts from faecal material in the environment or via ingested prey, it is suggested that as populations of humans and their companion animals grow, the impacts of faecal contamination on public and wildlife health are likely to increase [19]. Although the World Health Organisation advocate the collection of accurate epidemiological data on *T. gondii*[2] and the EU have recommended that all member states monitor *T. gondii* in animals entering the food chain [20], few countries regularly monitor its seroprevalence in humans, and still fewer focus on wildlife [2]. In the UK, the lack of national data on *T. gondii* in any wildlife species was explicitly highlighted in the recently published national risk profile for *T. gondii* commissioned by the Food Standards Agency, as a key knowledge gap with regard to protecting human health [16]. Opportunistic collection of wildlife provides a valuable opportunity to monitor a wide range of pathogens and contaminants (e.g. [21,22]), including *T. gondii*, and thereby offers a valuable opportunity to assess changing risk to human and animal health*.*

### Conclusions

We have confirmed that *Toxoplasma gondii* infection in Eurasian otters is widespread throughout England and Wales. Although there are no confirmed otter mortalities associated with *T. gondii* infection, given the ‘near threatened’ conservation status of the Eurasian otter and the link between *T. gondii* and mortality in sea otters [11], enhanced monitoring of otter health should be considered, that routinely includes serological investigation for *T. gondii* infection. Although it is not possible to discriminate between infection acquired from environmental sources (oocyst) or carnivorous activity (tissue cysts), the relatively high seroprevalence (29–51%) in all regions confirms that the risk of infection is significant across the country.

## Competing interests

The authors declare that there are no competing interests.

## Authors’ contributions

All authors contributed to study design and approved the final manuscript. EAC and DWF conceived the study, and EAC drafted the original manuscript. EAC, ESS, AC, SCP and CW performed otter post mortems and collated data. EG and JF analysed the serum samples. EFK, ESS and SCP conducted the statistical analyses. JC, SEP and EG made substantial contributions to study design, data interpretation, and revisions of the manuscript. All authors read and approved the final version of the manuscript.
